# Hypothyroxinemia in extremely low birth weight infants

**DOI:** 10.1186/1824-7288-41-S1-A16

**Published:** 2015-09-24

**Authors:** Paolo Ghirri, Francesca Dini, Sara Lunardi, Francesca Moscuzza, Antonio Boldrini

**Affiliations:** 1 Neonatology and Neonatal Intensive CareUnit and Section of Neonatal Endocrinology and Dysmorphology, S. Chiara Hospital, University of Pisa, Italy

## 

Hypothyroxinemia of prematurity (HOP) is a transient alteration in thyroid hormone availabilityfound in more than half of extremely low birth weight infants (ELBW) born at less than 30 weeks [1]. HOP is characterized by very low total T4 (TT4) and free T4 (FT4) levels with a normal or low thyroid stimulating hormone (TSH); TT4 and FT4 show a nadir at 7-10 days of life and they may remained low for the first 3-6 weeks of life depending from the severity of prematurity[2]. The reason for this hypothyroxinemia is multifactorial including the loss of maternal transfer of T4, immaturity of the hypothalamic-pituitary-thyroid axis, limited thyroid capacity to increase synthesis and metabolism, immaturity of peripheral tissue deiodination, reduced iodine availability and excessive losses, drugs affecting thyroid function and other adverse perinatal events [3].

Thyroid hormones are critical for a normal maturation of the developing brain in the fetus and infant, including cerebral neurogenesis, neural migration and differentiation, axonal and dendritic growth and synaptogenesis, gliogenesis, myelogenesis[4,5]. HOP has been associated with adverse neurodevelopmental outcomes such as deficits in developmental of motor, cognitive, language, memory, attention and it seem to be an independent risk factor for cognitive and behavioral deficits [6], however it is not clear the need to treat [7]. In a large trial of prophylacticthyroxine treatment van Wassenaer et al. [8]observed an 18-point improvement in mental development Bayley scores at 2 years postnatal age in infant born at less than 27 weeks gestation but, treated infants of more than 27 weeks had a 10-point deficit in Bayley scores. Overall there is insufficient evidence to determine whether use of thyroid hormones for treatment of preterm infants with transient hypothyroxinemia results in changes in neonatal morbidity and mortality, or reductions in neurodevelopmental impairments, but T4 supplementation may be beneficial in infants born at less than 27 weeks. 

Recently Scratch et al. [5], contrary to expectations, reported that in children born at less than 30 weeks’ gestation, higher concentrations of free thyroxine over the first 6 weeks of life were associated with poorer cognitive function at 7 years of age. Future studies should be directed in understanding unexpected endocrine patterns after very preterm birth. 
